# The psychological burden of an initially unexplained illness: patients with sternocostoclavicular hyperostosis before and after delayed diagnosis

**DOI:** 10.1186/1477-7525-8-97

**Published:** 2010-09-09

**Authors:** Willem A van der Kloot, Neveen AT Hamdy, Laurian CS Hafkemeijer, Femke MC den Dulk, Sadhna A Chotkan, Arnold AP van Emmerik, Ad A Kaptein

**Affiliations:** 1 Institute of Psychology, Leiden University, The Netherlands; 2 Department of Endocrinology and Metabolic Diseases, Leiden University Medical Center (LUMC), The Netherlands; 3 Department of Clinical Psychology, University of Amsterdam, The Netherlands; 4 Unit of Psychology, Leiden University Medical Center (LUMC), The Netherlands

## Abstract

**Background:**

Sternocostoclavicular hyperostosis (SCCH) is a rare, debilitating, chronic inflammatory disorder of the anterior chest wall due to a chronic sterile osteomyelitis of unknown origin. SCCH is largely underdiagnosed and often misdiagnosed. In individual cases it can remain unrecognized for years. The purpose of this study is twofold. Firstly, to evaluate the psychological condition of SCCH patients, both in the sometimes quite extended pre-diagnostic period between first manifestations and confirmed diagnosis of the disease, and in the current situation. Secondly, to investigate the relationships between the pre-diagnostic and the current psychological conditions of confirmed SCCH patients.

**Methods:**

Structured interviews were held with 52 confirmed SCCH patients. Questionnaires were included to assess posttraumatic stress symptoms, social support, aspects of pain, illness perceptions, self-reported health status, and quality of life.

**Results:**

SCCH patients reported stronger posttraumatic stress symptoms, more unfavorable illness perceptions, lower health status, and poorer quality of life than healthy individuals and patients with other diseases or traumatic experiences. Psychological distress in the pre-diagnostic period was associated with unfavorable conditions in the current situation.

**Conclusion:**

SCCH is an illness with serious psychological consequences. Psychological monitoring of patients with unexplained complaints is recommended as long as a diagnosis has not been reached.

## Background

Patients who suffer from rare diseases often encounter difficulties that victims of more common disorders are spared. Fewer health care providers have sufficient knowledge and experience to handle a rare disease and usually no or much fewer therapeutic options are available. Moreover, lack of awareness of the clinical manifestations of a rare disease may lead to diagnostic delay, failure of diagnosis, and misdiagnosis. Recently, these problems have been documented in a detailed report by the European Organisation of Rare Diseases, which was summarized in *The Lancet *[1,2]. In two surveys of a total of 12,000 patients with 16 different rare diseases, it was found that, with the exception of cystic fibrosis, 25% of the patients had to wait more than 3 years before the correct diagnosis was established, and that 41% were initially misdiagnosed, including 7% who were told that their symptoms were psychological or psychiatric. Eighteen percent of the patients had to find answers on their own to obtain the correct diagnosis. In 19% of the patients, diagnostic delay led to loss of confidence in the health-care system. Eighteen percent experienced rejection by a health-care professional because of disease complexity or associated symptoms.

The EURORDIS findings are paralleled by the results from a study of our own research group on patients with sternocostoclavicular hyperostosis (SSCH), a rare inflammatory disease of the axial skeleton [3]. Fifty percent of our patients had to wait between 3.5 and 36 years for a correct diagnosis, 40% were initially misdiagnosed, 4% were told that their symptoms were psychological, and 35% had sometimes felt rejected by doctors or nursing staff. In 23% the correct diagnosis was only established after the patients obtained information from sources outside their regular medical circuit.

SCCH is a rare chronic, inflammatory disorder of the axial skeleton, caused by a chronic sterile osteomyelitis with a predilection for the sternum, the medial ends of the claviculae, and the upper ribs. The most common clinical manifestations of SCCH include redness and (usually painful) swelling in the sternoclavicular region often associated with restricted and painful mobility of the adjacent shoulder(s). Thirty to fifty percent of the patients may develop pustulosis palmoplantaris, a chronic sterile inflammation characterized by sterile pustules on the palms of the hand or the soles of the feet, though not necessarily simultaneously with the bone manifestations.

SCCH was first described as a separate entity in 1974 in Japan and in 1975 in Germany [4,5]. In 1987, Chamot et al. coined the term SAPHO (Synovitis, Acne, Pustulosis, Hyperostosis, Osteitis) for a syndrome in which SCCH was associated with generalized joint and skin manifestations [6]. The literature shows that SCCH is an ill-known syndrome that may remain unrecognized for years [7-10] and that it is largely underdiagnosed due to a lack of awareness among physicians for the disorder [9-14]. Patients with SCCH have been described to go through a "diagnostic odyssey" [12, p. 209] before a correct diagnosis was obtained [15,16] and some dramatic examples were described of cases that were initially misdiagnosed and treated incorrectly [16,17]. Recently, we have shown that the duration of the interval between the first manifestations of SCCH and the establishment of its diagnosis had some serious negative psychological and socioeconomic consequences for SCCH patients [3].

### The pre-diagnostic period

During the often quite extended interval between the first manifestations of a rare disease and its correct diagnosis, patients live in a diagnostic vacuum as long as their symptoms remain medically unexplained and lack a descriptive diagnostic label. This situation, as Nettleton, Watt, O'Malley, and Duffey [18] demonstrated, made patients fear that their illness was "all in the mind" (p. 207) and "not legitimate" (p. 207). It made patients feel "marginalized by doctors" (p. 206), and made them represent their illness in terms of "chaos narratives" (p. 206) characterized by "confusion and uncertainty" (p. 206) and "a merry-go-round of hope and despair" (p. 206). Such strong feelings suggest that a prolonged pre-diagnostic period is stressful and traumatic and may produce serious repercussions on the mental health of the patients involved, for instance in the form of post-traumatic stress symptoms.

#### Post-traumatic stress

The relationship between post-traumatic stress (PTS) and physical illness has been documented in a variety of studies. PTS following violent or life-threatening experiences is often construed as a cause of or contributor to physical illness [19,20] although there is reason for caution with this interpretation [21]. Moreover, it has been observed that illness itself can be a traumatic condition that produces PTS. PTS was observed in women suffering from breast cancer in several stages [22-24], in patients after bone marrow transplantation for cancer [25], and among patients who underwent acute medical treatment in Intensive Care Units [26]. The latter study suggested that "the presence of traumatic *memories *[italics added] is one of the most relevant aspects for the development of PTSD-related symptoms" [26, p. 671]. Clinical levels of PTS were also found in women after spontaneous abortion [27], after abortion for fetal abnormality [28], after elective surgical abortion [29], and after childbirth [30,31]. For some women, the intense pain and feelings of helplessness during childbirth constitute a severely traumatic experience to which they may react with PTS [30,32]. In these cases (i.e. cancer, ICU-hospitalization, abortion, childbirth), it appears that the feelings and memories associated with the illness or violent bodily experience are the traumatic agents that produce or facilitate PTS.

In the interval between first symptoms and diagnosis, SCCH-patients experience pain, swelling, and/or restricted mobility of the shoulder girdle that are either not medically explained and thus remain untreated, or are incorrectly explained and consequently unsuccessfully treated. We hypothesized that in this situation, which may be worsened by feeling rejected by health professionals and others in the social environment, patients may develop symptoms of PTS.

#### Social support

Several studies have shown that coping with stress and traumatic events is positively influenced and moderated by the social support individuals receive or perceive [25,33-35]. Although some other studies [31,36] did not find an effect of social support on PTS, we hypothesized that social support will have a beneficial effect on PTS in SCCH-patients.

### The current situation

As SCCH is a chronic disease, a definitive cure cannot be achieved when the diagnosis is finally established. Treatment is mainly aimed at pain relief and control of the local inflammatory changes. However, even when the inflammatory manifestations are controlled, patients may retain some residual disability in the form of chronic pain and limited mobility of the shoulder girdle due to irreversible, degenerative changes in the structure of the affected bones. To better understand how SCCH may affect the life of patients with a confirmed SCCH diagnosis, we compared their quality of life (QOL), illness perceptions, and self-reported health status to those of patients with other diseases.

### Objectives

Our first objective was to evaluate the psychological condition of SCCH patients and the extent and seriousness of their problems both in the interval between first manifestations and diagnosis of the disease, and in the current situation, after the diagnosis had been established for some time. Our second objective was to investigate the relationship between the pre-diagnostic condition and the present-day situation of patients with SCCH. We expected that problems such as PTS in the pre-diagnostic period may have repercussions on QOL, illness perceptions, and self-reported health status at a later stage in the course of the illness. Moreover, because several studies have shown social support to have a positive effect on QOL [37,38], we tested the hypothesis that social support in the pre-diagnostic period is related to (perceived) physical and mental health at the time of the study.

## Materials and methods

The study was initiated and conducted by the Psychology Institute of Leiden University. This institute does not require ethical approval and does not comprise an ethical commission. Design and execution of the study followed the ethical standards of the American Psychological Association. The study was supported by the Dutch SCCH Patient Association http://www.scch.nl, which helped to recruit the majority of patients from its members. Most patients were also under regular clinical control of one of the authors (NATH) at the Department of Endocrinology and Metabolic Diseases of the Leiden University Medical Center (LUMC), a tertiary referral center for patients with SCCH.

### Patients

Fifty-seven patients with a definitive diagnosis of SCCH were invited to participate in this study. Fifty-two of them responded positively and were interviewed in their homes by one of three trained interviewers: two psychology Master students and one final-year medical student. We estimated that these 52 respondents encompassed the majority of Dutch patients with a diagnosis of SCCH and were representative of this population. The patients were contacted by telephone and if they agreed to participate, a date and time were set for the interview. Written informed consent was obtained from all patients prior to the start of the interviews.

### Interviews

Interviews consisted of a structured set of questions on gender, age, age at the time of the first manifestations of SCCH, education, employment, and several aspects of the patient's medical and psychological condition both in the pre-diagnostic period and in the present situation. The patients were also interviewed about their trajectories in the health care system between onset of the disease and confirmed diagnosis (those results have been published elsewhere [3]).

The interviewers asked most questions verbally. The responses were written on protocol sheets and (after permission) were registered on a voice recorder. During the interview the respondents also completed several questionnaires. To describe their condition in the *pre-diagnostic period*, the patients were asked to localize their complaints on drawings from the Dutch language version of the McGill Pain Questionnaire (MPQ-DLV) [39] and were presented with Dutch versions of the Revised Impact of Event Scale (IES-R) [40] and the Social Support Inventory (SSI) [41]. With regard to their *present situation*, the patients filled out the complete MPQ-DLV, the Dutch version of the Brief Illness Perception Questionnaire (B-IPQ) [42], and the MOS Short-Form General health Survey (SF-20) [43].

### Questionnaires

The IES-R measures symptoms of PTS by means of 22 statements (e.g. "In the period when it was not yet clear that my complaints were due to SCCH, I found it difficult to concentrate") with four response categories "never", "rarely", "sometimes", and "often", which, using the original scoring system, were coded as 0, 1, 3, and 5. From these items scores were computed on the three subscales intrusion, avoidance, and hyperarousal. These subscales were subsequently combined to form the IES-R-total score.

Five emotional impact statements ("my complaints made me *desperate*", "my complaints felt like a *drama*", "I was very *concerned *about my complaints", "I was very *frightened *about my complaints", and "I was very *depressed *about my complaints") were presented with response categories 0 = never, 1 = rarely, 2 = sometimes, and 3 = often.

The SSI contains 20 questions (e.g. "In the period when it was not yet clear that your complaints were due to SCCH, how often did somebody cheer you up?") with response categories (1) much too infrequently, (2) somewhat too infrequently, (3) just right, (4) somewhat too often, and (5) far too often. The 5 and 4 responses were recoded into 1 and 2, respectively, yielding scores that measure *satisfaction *with support. Four subscales were computed: emotional support, informative support, instrumental support, and social companionship, which also were aggregated into SSI-total.

The MPQ-DLV consists of four parts. The first part shows two drawings of a nude, androgynous person, seen from the front and from the back, on which the respondents indicated the regions where they felt pain or had other complaints. The drawings were scored by superimposing a 0.5 × 0.5 cm grid and counting the number of grid cells marked by the respondent. This resulted into four variables: frontal-complaints-first and dorsal-complaints-first concerning the first, pre-diagnostic, manifestations of SCCH, and frontal-complaints-now and dorsal-complaints-now for the present situation. The second part consists of questions regarding pain from which we selected "Do you ever feel pain?" with response categories (1) never, (2) yes, but the pain comes in waves and disappears between the waves, (3) yes, the pain is always present but its intensity varies, and (4) yes, the pain is always present and has always the same intensity. The third part consists of two 100 mm visual analogue scales (VAS) with endpoints "no pain at all" and "unbearable pain". On the first VAS, the respondents rated the intensity of their current pain (VASnow). On the second VAS they rated the intensity of their pain when it was least intense (VASmin) and when it was most intense (VASmax). The last part of the MPQ-DLV consists of 20 sets of three or four pain describing adjectives. In each set that contained adjectives that were applicable to their pain, respondents marked the one adjective that best described their pain. The sets of adjectives describe three different qualities of pain: sensory (e.g. burning, sharp, pinching), affective (e.g. exhausting, fearful), and evaluative (e.g. annoying, unbearable). From the marked adjectives four pain rating indices (PRIS, PRIA, PRIE, PRIT) were constructed that indicate the intensities of the sensory, affective, and evaluative qualities of pain, respectively, as well as the total intensity.

The B-IPQ contains eight questions (e.g. "How long do you think your illness will continue?") each measuring a perception of one's illness. The perceptions (called dimensions) are labeled consequences, time-line, personal control, treatment control, identity, concern, understanding, and emotional response. Each response is measured on a scale from 0 to 10 with the appropriate labels.

The SF-20 contains 20 questions that concern health status and quality of life. The items (e.g. "Are you restricted in bending, lifting, or stooping?") are summarized by six dimensions: physical functioning, role fulfillment, social functioning, mental health, perceived health, and physical pain. Higher scores indicate more favorable conditions.

### Analysis

Two of the authors categorized the responses to the open questions. Interobserver agreement was "good" to "perfect" with Kappa's between 0.70 and 1.00 (quartiles: 0.83, 0.94, and 0.98). For the questionnaire data, Cronbach's alphas, means, and standard deviations (SD) were computed. Means of the present sample were compared with means obtained from samples in other studies, using analysis of variance (ANOVA) and Bonferroni comparisons. Relationships among variables were studied by means of Pearson product moment correlations and various multivariate techniques, notably, multiple regression, principal component analysis, and canonical correlation analysis.

## Results

The respondent group consisted of 46 women (88.5%) and 6 men (11.5%) whose ages varied between 24 and 79 years (median: 56 yrs). The oldest complaint dated back 49 years, the most recent one 2 years (median 11 yrs). The age at which the first complaints arose (age-first) ranged from 15 to 72 years (median 41.5 yrs). The time interval between the patient's first consultation and establishment of the diagnosis varied from 1 month to 36 years. In 4 cases, the complaints started before 1976, that is, earlier than, or at the same time as the first publications on SCCH; therefore those patients could not possibly have been diagnosed before 1976. For those patients we have used 1976 as the year of first consultation. The maximum interval length then becomes 24 years with a mean of 5.6 ± 5.9 years and a median of 3.5 years. The 25% quartile was exactly 1 year; the 75% quartile was equal to 8.75 years. The earliest SCCH diagnosis among our respondents was established in 1988. The most recent diagnosis was confirmed in 2007, the year our study was run. The median was reached in 2000; the 25% and 75% quartiles were in 1995 and 2003, respectively

### The pre-diagnostic interval

The frontal complaints at first manifestation of SCCH ranged from 0 to 32 marked grid-cells (possible range: 0 - 111; mean = 4.58; SD = 6.65). The dorsal complaints at first manifestations ranged from 0 to 50 (possible range: 0 - 134; mean = 3.15; SD = 8.65). Sixty-one percent of the frontal and 59% of the dorsal markings, respectively, were in the upper thoracic and shoulder regions.

Table 1 contains the frequencies and percentages of the responses to the open questions concerning the period between first symptoms and definitive diagnosis of SCCH. The majority of the respondents were seriously affected by their disease, as 86.5% was no longer able to perform their normal work or other activities, and 88.5% felt worried, depressed, or misunderstood. Although 78.9% of the patients reported support from their environment, at least from their close relatives, 48.1% experienced that others had doubts about their illness and 32.6% reported that others believed their illness to be psychological or a form of affectation. Moreover, 21.2% of the patients *themselves *sometimes doubted they were really ill and thought their complaints might be psychological. Some forty percent of the respondents felt guilty, of whom 34.6% often. Two-thirds (67.3%) reported that they (sometimes or often) had not been taken seriously by their health-care providers, among whom their own general practitioners. These data confirm all the major themes (uncertainty, despair, guilt, "it is all in the mind", marginalization by doctors) that were found among the patients with an unexplained illness in the study by Nettleton, et al. [18].

**Table 1:** Frequencies and percentages of coded responses to the open questions concerning the pre-diagnostic interval.

During the interval before diagnosis, were you *able to carry on *with your normal work and other activities?		
No	45	86.5%
Yes	7	13.5%
How did you *feel *during the interval before diagnosis?		
Worried, frightened	25	48.1%
Depressed, desperate, overwhelmed by disease	12	23.1%
Misunderstood, not taken seriously, angry	9	17.3%
Normal, matter of fact	6	11.5%
How did your *environment react?*		
Worried, startled	15	28.9%
Helpful, empathic, supportive	21	38.5%
Empathy at first, later less	2	3.9%
Normal, no empathy, unbelief	10	19.2%
Patient did not tell environment about disease	4	7.7%
Did your *environment support *you?		
Never	3	5.8%
Sometimes, little support	5	9.6%
Gave much support	24	46.2%
Support only from close family	17	32.7%
Patient did not need support	3	5.8%
What was the *extent of the received support?*		
No support	6	11.5%
Little support, sometimes support	13	25.0%
Much support	33	63.5%
Did *others doubt *that you were ill?		
Never, always taken seriously	23	44.2%
No doubt in close environment, rest doubted	12	23.1%
Doubt, did not think complaints were important	13	25.0%
Patient did not consider herself ill	1	1.9%
Environment did not know about disease	3	5.8%
Did you *yourself *sometimes *doubt *that you were ill?		
No	43	82.7%
Sometimes	9	17.3%
Did *others *think your complaints were psychological, "all in the mind"?		
No	35	67.3%
Yes, disease was psychological	15	28.8%
Affectation	2	3.8%
Did *you yourself *think that your complaints were psychological, "all in the mind"?		
No	41	78.8%
Sometimes	11	21.2%
Did you sometimes feel *guilty *about your illness?		
No	31	59.6%
Sometimes	3	5.8%
Often	18	34.6%
Were you sometimes *not taken seriously *by health-care providers?		
No, always taken seriously	17	32.7%
Yes, by General Practitioner (GP)	13	25.0%
Yes, by some doctors (GP or specialist)	19	36.5%
Yes, by hospital staff	3	5.8%
*How often *were you *not taken seriously *by health-care providers?		
Never	17	32.7%
Sometimes	27	51.9%
Often	8	15.4%

The five statements "*desperate*", "*drama*", "*concerned*", "*frightened*", and "*depressed*", which could be endorsed on a scale from 0 (never) to 3 (often), had means and SDs of, respectively, 1.4 ± 1.19, 1.29 ± 1.09, 1.71 ± 1.05, 1.08 ± 1.05, and 1.65 ± 1.08. The mean and SD of the scales after summation was 7.13 ± 4.78. The Cronbach Alpha reliability coefficient was .922. Thirteen (25%), eight (15.4%), thirteen (25%), five (9.6%), and thirteen (25%) respondents gave the response "often" to the five questions. These results indicate that having an (initially) unexplained illness has serious psychological mental consequences for a substantial number of patients.

#### PTS symptoms

Table 2 contains the means, SDs and ranges of our respondents' scores on the three subscales and the total scale of the IES-R. For comparison we have added the means and SDs of two other Dutch samples: 435 women who recently gave birth [32] and 191 women who had their pregnancies terminated because of fetal abnormality [28].

**Table 2:** Mean ± SD, number of items, range of observed scores, Cronbach's α, and *F*-values of the subscales of the Impact of Event Scale (Revised) observed in the present sample and in two comparison samples (means with equal upper case superscripts are not significantly different)

IES-R scale (# of items; range; α)	SCCH patients n = 52	Childbirth data^a^ n = 453	Pregnancy termination data^b^ n = 191	*F*-value *df *= 2, 675
Intrusion (8; 0 - 40; .866)	16.12^A ^± 9.76	7.24^C ^± 6.8	9.35^B ^± 8.02	34.959**
Avoidance (8; 0 - 36; .854)	16.83^A ^± 9.69	2.13^C ^± 4.2	5.64^B ^± 7.54	153.220**
Hyperarousal (6; 0 - 30; .843)	12.50^A ^± 8.27	3.23^C ^± 4.3	4.78^B ^± 6.46	70.036**
Total (22; 2 - 96; .931)	45.45^A ^± 24.73	12.61^C ^± 13.0	19.78^B ^± 19.48	99.230**

^a^Olde et al. [32]; ^b^Korenromp et al. [28]; ** = *p *< .0001.

ANOVAs showed significant overall differences (see Table 2) among the means of the different samples. In order to study differences among the means of the separate samples, we performed 3 post hoc Bonferroni comparisons for each scale using α = .05/3 (two-tailed) and critical *t*-value = ± 2.401 (*df *= 675). On all IES-R scales and IES-R-total, the means of the scores of SCCH patients were significantly greater (p < .0001) than those of the other groups.

#### Social support

Table 3 contains the means and SDs of the SCCH patients on the scales of the SSI. Where the possible scores of the SSI subscales range from 5 to 15 and the SSI-total from 20 to 60, the observed means of the SCCH patients are located in the upper regions of the possible scores, which means that our respondents have felt on average rather satisfied with the experienced degree of social support. This corroborates the data on support in Table 1. As we could not find means and SDs of other samples in the literature, we cannot compare our SSI data with those of other studies.

**Table 3:** Mean ± SD, number of items, observed and possible range, Cronbach's α, and correlations with hyperarousal and IES-R-total of the Social Support Inventory scales

					Correlations with	
					
Scale name	Mean ± SD	Number of items	Range of scores^a^	Cronbach's α	hyperarousal	IES-R-total
Emotional support	12.70 ± 2.43	5	5 - 15	.842	-.294 (.018^b^)	-.250 (.039^b^)
Instrumental support	12.02 ± 2.60	5	5 - 15	.742	-.380 (.003^b^)	-.265 (0.30^b^)
Social companionship	12.17 ± 2.85	5	5 - 15	.844	-.288 (.020^b^)	-.206 (.074^b^)
Informative support	12.39 ± 2.54	5	5 - 15	.782	-.236 (.048^b^)	-.166 (.123^b^)
SSI total	49.29 ± 9.10	20	20 - 60	.931	-.343 (.007^b^)	-.253 (.037^b^)

^a^The ranges of the observed scores were equal to the ranges of the possible scores. ^b ^One-tailed probability.

To test the - directional - hypothesis that social support has a negative association with PTS symptoms, we computed bivariate correlation coefficients between the four IES-R scales and the five SSI scales and used one-tailed tests of significance. All SSI scales had significant negative correlations with hyperarousal and three SSI scales (SSI-emotional, SSI-instrumental, and SSI total) had significant negative correlations with IES-R total (see Table 3). The latter three correlations are explained by the correlations between the SSI scales and hyperarousal, as all other coefficients were not significant. Thus, it appears that hyperarousal (i.e. irascibility, hypervigilance, concentration and sleeping problems) is tempered by all four modes of social support. However, all correlation coefficients are rather small. The SSI scale with the highest correlation (SSI instrumental) explains only14.4% of hyperarousal; this amount cannot be increased by adding the three other SSI scales as predictors, because they are highly collinear (see below).

Principal component factor analysis of the three IES-R subscales and the subscales of the SSI yielded two factors that together explained 78% of the variance. After Oblimin-rotation, one factor coincided with the three IES-R subscales and the other with the SSI subscales. Factor scores of the two factors completely coincided with, respectively, IES-R-total (*r *= .999) and SSI-total (*r *= 1.000). The two oblique factors were almost perpendicular, with a correlation of -.244 (6% common variance), which is barely significant at α = .05, one-tailed. Therefore, the hypothesis that social support has a beneficial effect on PTS is only weakly supported in the present study.

Product-moment correlations were computed between age-first, frontal-complaints-first, dorsal-complaints-first, and diagnostic delay, on the one hand, and the four IES-R and five SSI scales on the other hand. None of the correlations with the IES-R scales were significant. Age at first manifestations and diagnostic delay correlated significantly with emotional support (resp. *r *= .374, *p *= .007; *r *= - 358, *p *= .010) and with social companionship (resp. *r *= .327, *p *= .019; *r *= -.347, *p *= .013). Diagnostic delay also had significant correlations with SSI informative support (*r *= -.342, *p *= .014) and SSI total (*r *= -.356, *p *= .010). The positive correlations between age-first and the two SSI scales, might be explained by the fact that the respondents who fell ill at an older age had a more elaborate social network (e.g. spouse and children) to provide support. The negative correlations between diagnostic delay and the three social support scales could mean that social support is relatively short lived: initially strong, it probably wanes with the persistence of unexplained complaints.

#### Reaction to diagnosis

Responses to the interview questions "Did something change for you when it was established with certainty that you had SCCH?" and "Did you feel better or worse than before the diagnosis?" overlapped and were therefore combined. Twelve patients (23.1%) felt better because "now there was clarity, it had a name, it was real", 11 patients (21.2%) felt relief because treatment was possible and they could make adaptations, 5 patients (9.6%) felt relief because it was nothing more serious such as cancer or rheumatoid arthritis, 9 patients (17.3%) reported ambiguous feelings: they felt relief because of more clarity but also realized they had a chronic disease. Three patients (5.8%) said they felt worse after the diagnosis was established, 2 patients (3.9%) reported no change at all, and 10 (19.2%) gave ambiguous answers containing positive and negative elements.

### Current condition

#### Clinical symptoms

On the frontal-complaints-now drawing of the MPQ-DLV, the patients marked on average 7.12 (SD = 9.43) grid cells; on the dorsal-complaints-now variable the mean was 5.23 (SD = 8.04). In both cases these means were significantly higher (*t *= 2.152, *p *= .036 and *t *= 2.009, *p *= .05; *df *= 51, resp.) than the means on frontal-complaints-first and dorsal-complaints-first, suggesting a deterioration of symptoms over time.

Four patients (7.7%) reported they never had pain, 13 patients (25%) had pain that came in waves with symptom-free periods, in 32 patients (61.5%) the pain was continuous but of variable intensity, and in 3 patients (5.8%) pain was continuous with no difference in intensity. Mean scores and SDs of the visual analogue scales for present pain (VASnow), minimum pain (VASmin), and maximum pain (VASmax) were 35.50 ± 27.41, 18.54 ± 17.87, and 74.33 ± 25.87, respectively. These means, although higher, do not differ significantly from those of a Dutch sample [44] of 227 persons receiving physiotherapy for mobility problems (VASnow = 29.43 ± 22.64, VASmin = 15.36 ± 14.37, VASmax = 67.17 ± 23.82). In the present study, pain rating indices for the sensory (PRIS), affective (PRIA), and evaluative qualities (PRIE) of pain had means and SDs of 9.29 ± 6.96, 3.46 ± 3.05, 4.27 ± 2.58. The mean of the total pain rating index (PRIT) was 17.02 ± 11.40. Conservative *t*-tests using *df *= 51 showed that all means were significantly higher (*p*-values.001, .0006, .021, and .0006, respectively) than the means of the patients undergoing physiotherapy reported in [44] (PRIS: 5.80 ± 4.08; PRIA: 1.83 ± 1.97; PRIE: 3.37 ± 1.74; PRIT: 11.00 ± 7.03). These data suggest that the pain experienced by SCCH patients is similar or worse than the pain of the general, heterogeneous, population of individuals with usually painful mobility complaints who are treated by physiotherapy.

#### Limitations and functioning

Nineteen respondents (36.5%) held jobs, 14 of whom (26.9%) part-time. Ten respondents (19.2%) were unemployed, 10 (19.2%) were retired, and 13 patients (25%) were on social benefits for permanent disability (regulated by Dutch national law). Among the 42 SCCH patients who were either unemployed, worked part-time, or were on social benefits, 22 (42.3% of the total) attributed this to their disease. This indicates that in a substantial number of cases SCCH is associated with debilitating limitations that affect the socio-economic situation of patients.

Table 4 contains the means, SDs, ranges, and Cronbach's alphas of the subscales of the SF20. Also shown are the means and SDs reported by Sonino et al. [45] of 86 patients with various forms of pituitary disease and 86 healthy respondents used as controls. ANOVAs yielded significant overall *F*-values (Table 4). Bonferroni comparisons using α = .05/3 showed that all groups differed significantly from each other as regards physical functioning, health perceptions, and pain, with the SCCH patients having the least favorable scores. Regarding role functioning, the SCCH patients did not differ significantly from patients with pituitary disease but had significantly lower means than the healthy control group. The same pattern of results was obtained for social functioning and mental health. In sum, all means of the SCCH patients were significantly lower than those of the healthy group, and consistently lower (though not always statistically significant) than those of the pituitary patients.

**Table 4:** Mean, SD, number of items, observed range, Cronbach's α, and *F*-values of the SF20 scales observed in the present study and among patients and healthy controls in a comparison study (means with equal upper case superscripts are not significantly different).

SF20-scale (# of items; observed range; α)	SCCH patients n = 52	Pituitary disease		ANOVA *F*-value; *df *= 2, 221
			
		**Patients**^**c**^ n = 86	**Controls**^**c**^ n = 86	
Physical functioning^a^ (6; 6 - 12; .673)	8.44^A ^± 1.58	26.6^B ^± 3.7	28.6^C ^± 2.1	983.36 **
Role functioning^a^ (2; 2 - 4; .766)	2.46^A ^± .75	2.8^A ^± 1.5	3.7^B ^± .7	25.237 **
Social functioning^a^ (1; 1 - 6; --)	4.33^A ^± 1.21	4.8^A ^± 1.7	5.7^B ^± .7	20.948 **
Mental health^a^ (5; 5 - 30; .933)	21.61^A ^± 5.13	22.8^A, B ^± 5.9	24.6^B ^± 3.6	6.417*
Health perceptions^a^ (5; 5 - 30; .745)	14.08^A ^± 3.91	19.6^B ^± 4.3	22.8^C ^± 2.4	95.857 **
Pain^b^ (1; 1 - 5; --)	3.63^A ^± 1.27	2.1^B ^± 1.2	1.7^C ^±.8	53.909**

^a^Higher values indicate better functioning and health. ^b ^Higher values indicate more pain. ^c^Sonino et al. [45], the data of the Sonino et al. study were transformed to make them comparable to the means of the SCCH study. * p < .002. ** p < .00001.

#### Illness perceptions and disease acceptance

Table 5 contains the means and SDs of the SCCH patients on the eight dimensions of the B-IPQ, as well as the means of two groups of patients (with type 2 diabetes and asthma) which were selected for comparison from a study by Broadbent et al. [46]. Type 2 diabetes and asthma are chronic conditions that can be relatively well-controlled by medication and personal life-style, though diabetes may have serious long-term complications. ANOVAs showed significant overall differences (Table 5) among the three patient groups on all B-IPQ variables except timeline. The latter result suggests that all groups were equally aware of the chronic nature of their diseases. Bonferroni comparisons with α = .05/3 showed that as regards consequences (i.e. the perceived impact of one's illness) SCCH patients had significantly higher means than asthma patients but did not differ from patients with diabetes. A possible explanation is that diabetes and SCCH are conditions that are continuously present, whereas asthma usually manifests itself in acute exacerbations that are spaced in time. SCCH patients perceive lower personal and treatment control, and experience more physical complaints (the dimension "identity") than patients with asthma and diabetes. This probably reflects the fact that there is as yet no standard treatment for SCCH and that various applied treatments are only partially or not effective in controlling symptoms or disease progression. Life-style appears to have limited influence on symptomatology in SCCH. Regarding coherence, concern, and emotional response, the SCCH patients did not differ from patients with asthma, but both groups had significantly lower means than the diabetes patients. This may be due to the fact that diabetes is a much better understood disease, treatment is well-established, and patients are well aware of its possible serious consequences if left untreated.

**Table 5:** Means and SDs on the eight B-IPQ dimensions and two comparison samples (means with equal upper case superscripts are not significantly different).

	SCCH patients	Asthma patients^a^	Diabetes 2 Patients^a^	*F-value *(*p*)
B-IPQ scale	n = 52	n = 309	n = 119	*df = *2, 477
Consequences	5.63^A ^± 2.80	3.5^B ^± 2.3	4.7^A ^± 2.9	21.575 (< .0001)
Timeline	9.17 ± 1.86	8.8 ± 2.2	9.2 ± 1.9	1.920 (.1478)
Personal control	5.31^A ^± 2.35	6.7^B ^± 2.4	6.7^B ^± 2.3	7.9729 (.0004)
Treatment control	6.8^A ^± 2.37	7.9^B ^± 2.0	8.0^B ^± 2.3	6.660 (.0014)
Identity	6.21^A ^±2.30	4.5^B ^± 2.3	4.6^B ^± 2.8	11.153 (< .0001)
Concern	4.77^A ^± 3.26	4.6^A ^± 2.8	7.0^B ^± 3.1	2.520 (< .0001)
Coherence	6.15^A ^± 2.54	6.5^A ^± 2.6	7.9^B ^± 2.3	15.222 (< .0001)
Emotional response	4.04^A ^± 3.21	3.3^A ^± 2.9	4.3^B ^± 3.3	5.192 (.0059)

^a ^Broadbent et al. [46].

The patients answered several open questions concerning the acceptance for their illness by themselves and their social environment. Twenty-two patients (42.3%) said they accepted their disease, 16 (30.8%) accepted their disease but found it difficult to do so, and 12 (23.1%) did not accept that they were ill. Two patients (3.9%) gave contradictory responses. Twenty-four patients (46.2%) considered it difficult to live with SCCH, in 22 cases (42.3%) because of the limitations caused by the illness and in two cases (3.8%) because of anxiety and concern about the future. Twenty-eight patients (53.8%) did not find it difficult to live with SCCH, 23 (44.2%) because they had learnt to adapt to their illness and 5 (9.6%) because treatment was effective. Thirty-four respondents (65.4%) reported that they experienced sufficient understanding for their illness in their environment, whereas 14 (26.9%) reported insufficient understanding and 4 (7.7%) felt only understood by their partner and the persons nearest to them. Thirty-six respondents (69.2%) experienced no problems with the reactions of their environment; 4 patients (7.7%) had experienced such problems in the past, and 12 (23.1%) experienced problems in the present situation. Twelve respondents (23.1%) had experienced problems in their jobs, with finding new jobs, or getting functions adapted to their limitations. Nine patients (17.3%) had difficulties obtaining insurance or applying for social benefits.

### Relationships between pre-diagnostic and current conditions

As the variables describing the pre-diagnostic condition and those concerning the present-day situation are numerous and internally collinear, data reduction was necessary before we could study their relationships meaningfully. Above, we already reported that the IES-R scales intrusion, avoidance, and hyperarousal can be summarized by their total (IES-R-total) and that the SSI scales can be represented by SSI-total without significant loss of information. In a similar way (i.e. principal component analysis) we found that the rating scales measuring despair, drama, concern, fright, and depression could be reduced to one factor that explained 76.4% of their variance. Scores on this factor were perfectly correlated with the sum of the five variables. Therefore, we have used this sum in our final analyses, and named it depressed-total, because depressed had the highest loading.

The SF20 subscales and the B-IPQ dimensions were submitted to principal component analysis and hierarchical cluster analysis to explore which scales could be joined. All SF20 scales except SFpain could be aggregated into one summary variable. We therefore computed the total of the first five SF20 subscales and labeled this new variable SF-general-health.

Among the B-IPQ dimensions, one new variable (BIPQ-impact) was constructed by summing consequence, identity, concern, and emotional response, as these variables had extensive communalities. We also summed the dimensions treatment control and coherence. As the latter variables suggest understanding of the disease, we labeled their sum BIPQ-cognition. Because timeline and personal control showed less association with the other BIPQ dimensions they were used separately in the analyses.

We performed multiple regression analyses with age-first, frontal-complaints-first, dorsal-complaints-first, IESRtotal, SSItotal, depressed-total, and diagnostic delay as the predictors of frontal-complaints-now, dorsal-complaints-now, VASnow, VASmin, VASmax, PRIS, PRIA, PRIE, BIPQ-cognition, timeline, personal control, BIPQ-impact, SFtotal, and SFpain, respectively. The results, listed in Table 6, show that all current condition variables except personal control can to some extent be predicted by one or more pre-diagnostic variables with statistical significance. Our data also show that all pre-diagnostic variables, except IES-R-total, contribute to one or more of the predictions. The most important predictors (in terms of number of contributions) are SSI-total, Age-first, Depressed-total, and dorsal-complaints-first. SSItotal has positive contributions to variables indicating favorable conditions (SFtotal and BIPQcognition) and contributes negatively to variables that express unfavorable aspects (frontal-complaints-now, dorsal-complaints-now, VASnow, PRIA, and SFpain). Depressed-total has positive relations with the (unfavorable) VASmax, PRIA, and BIPQimpact, whereas it contributes negatively to the (favorable) SFtotal. The contributions of age-first and dorsal-first, are, however, more difficult to understand.

**Table 6:** Multiple regression coefficients and standardized regression weights for predicting variables representing present-day conditions from variables representing pre-diagnostic aspects.

	Pre-diagnostic variables (predictors)							
	
Present-day variables (dependents)	Age-first	Frontal-first	Dorsal-first	IESR-total	SSI-total	Depressed-total	Delay	R
Frontal-now^a^	-	.304*	.323*	-	-.355**	-	-	.647**
Dorsal-now^b^	-	.	. 627**	-	-.428**	-	-	.738**
VASnow^c^	-	-	-	-	-.296*	-	-	.296*
VASmin^d^	-	-	-	-	-	-	.413**	.413**
VASmax^e^	-	-	-	-	-	.418**	-	.418**
PRIS^f^	-.428**	-	-	-	-	-	-	.428**
PRIA^g^	-	-	.255*	-	-.252*	.543**	-	.722**
PRIE^h^	-.355*	-	-	-	-	-	-	.355*
SFpain^i^	-	-	-	-	-.284*	-	-	.284*
SFtotal^j^	-	-	-	-	.387**	-.404**	-	.653**
BIPQimpact^k^	-.243*	-	-	-		.619**	-	.730**
BIPQcognition^l^	-.317*	-	-.447**	-	.332*	-	-	.510**
Timeline	-.369**	-	-	-	-	-	-	.369**
Personal control	-	-	-	-	-	-	-	-

^a^Frontal-now = frontal area indicated as presently painful; ^b^Dorsal-now = dorsal area indicated as presently painful; ^c^VASnow = visual analogue scale score of present pain; ^d^VASmin = visual analogue scale score of minimal pain; ^e^VASmax = visual analogue scale score of maximal pain; ^f^PRIS = MPQ pain rating index (sensory); ^g^PRIA = MPQ pain rating index (affective); ^h^PRIE = MPQ pain rating index (evaluative); ^i^SFpain = pain score measured by SF20; ^j^SFtotal = total of SF20 subscales physical functioning, role fulfillment, social functioning, mental health, and perceived health; ^k^BIPQimpact = sum of BIPQ consequence, identity, concern, and emotional response; ^l^BIPQcognition = sum of BIPQ treatment control and coherence.

To clarify the above results, we performed a canonical correlation analysis (CANCOR) by means of SPSS MANOVA [47-49]. CANCOR investigates the relationships between two sets of variables by simultaneously looking for principal components in both sets under the restriction that the correlation between the first component in Set 1 and the first component in Set 2 is maximized. The same is required of the subsequent dimensions. The principal components (also called canonical axes or dimensions) can be interpreted by means of their loadings, that is, the correlations between the components and the original variables. Interpretation is often enhanced by graphs in which the components are represented by orthogonal dimensions and the variables by arrows whose coordinates on the dimension are given by the loadings.

**Figure 1:**
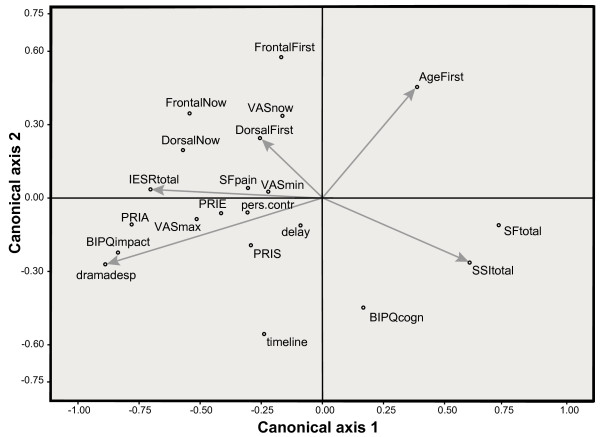
**Loadings of pre-diagnostic variables and present-day variables on two canonical axes**. Each variable is a vector in the canonical space; for legibility we have only drawn vectors for the pre-diagnostic variables. Vector lengths indicate their importance in the relationship between the two sets of variables.

In our case, the first set of variables consisted of age-first, frontal-complaints-first, dorsal-complaints-first, SSItotal, IES-R-total, depressed-total, and diagnostic delay. The second set contained frontal-complaints-now, dorsal-complaints-now, VASnow, VASmin, VASmax, PRIS, PRIA, PRIE, BIPQ-cognition, timeline, personal control, BIPQ-impact, SFtotal, and SFpain. CANCOR yielded two components (canonical axes) with statistically significant correlations (*r*_1 _= .911, *p *< .001; *r*_2 _= .830, *p *< .017). Figure 1 displays the loadings of the variables on the canonical axes.

The loadings of the current condition variables on the horizontal axis suggest that this dimension reflects apperceptions of health because the rightmost end is defined by SFtotal, i.e. general perceived health (higher scores denote better health), and the leftmost end by BIPQ-impact and PRIA, which measure negative affective reactions to illness and pain. The lower end of the vertical axis is defined by BIPQ-cognition and timeline. This possibly indicates that individuals with a better understanding of their illness and its treatment have clearer ideas about the chronic character of SCCH. Those individuals have currently lower scores on VASnow and frontal- and dorsal-complaints-now.

The loadings of the pre-diagnostic variables confirm our interpretation of the horizontal axis: its left extreme is defined by depressed-total and IES-R-total, variables which measure strong affects. Thus, strong negative feelings in the pre-diagnostic period are associated with strong negative affects at the time of the interview. The high positive loadings of SSI-total on Axis 1 show that social support may counteract such negative affects. The direction of SSI-total explains its role in the regression analyses: it runs into the same direction as the favorable SFtotal and BIPQcognitions, and in the opposite direction of the unfavorable frontal-now, dorsal-now, VASnow, and PRIA. Depressed-total has high loadings on the unfavorable end of Axis 1, which explains its positive weights in the prediction of BIPQimpact, PRIA, and VASmax and its negative weight in predicting SFtotal.

Although IES-R-total loads highly on the negative end of Axis 1, it played no role in any of the regression analyses. Figure 1 shows why: IES-R-total, depressed-total, and SSI are strongly interrelated (collinear) and cannot therefore be entered together in a regression analysis. The larger loadings of SSI-total and depressed-total explain their precedence over IES-R-total.

The second axis has high loadings of frontal-complaints-first and age-first, that is, patients with more anterior (chest wall) complaints and those who were older at the onset of SCCH tend to have higher scores on VASnow and on frontal-complaints-now at a later date. Interestingly, frontal-complaints-now and dorsal-complaints-now have higher (negative) loadings on the first axis than on the second axis. This means that these 'objective' indicators of SCCH in the present situation are also associated with affective reactions in the past.

## Discussion

The above results consistently indicate that the average patient with SCCH experienced serious psychological problems both in the current situation at the time of the interview and in the pre-diagnostic period between first manifestations of the disease and establishment of the diagnosis of SCCH. With respect to the pre-diagnostic period, patients reported affect and behavior that classify as PTS symptoms. Comparisons with reference groups made it clear that SCCH patients had significantly higher scores on measures of PTS than women who had experienced pregnancy termination because of fetal abnormality, and women three months after childbirth [28,32]. We speculate that the traumatic experiences of having an unwanted abortion, however sad, or giving birth, however violent, have a shorter duration than being victim of an as yet unexplained and thus untreatable illness, which can be construed as a chronic stressor. It should be noted here, that the IES-R questionnaire we used, measures PTS *symptoms*, and does not yield a formal PTSD diagnosis. Although some authors would argue that serious physical illness does not meet the current DSM-IV definition of a traumatic event, the present study is in line with others that have documented elevated levels of PTS after breast cancer [22-24], bone marrow transformation for cancer [25], and acute medical treatment in Intensive Care Units [26]. Regardless of the exact demarcation of the trauma concept, the present study demonstrates that our SCCH patients reported elevated levels of intrusion, avoidance, and hyperarousal symptoms in response to their illness.

SCCH patients reported fair to good social support during the pre-diagnostic period. We found that support was greater in patients who were older during the pre-diagnostic interval, which is possibly explained by the fact that older persons have more stable and more intimate social networks, which stood the test of time. We also found that social support was inversely related to diagnostic delay, indicating that this form of support may diminish as the unexplained illness lasts longer. Our expectation that social support would have a beneficial effect on PTS was indeed confirmed, although the correlation found was very weak.

SCCH patients reported equal or more pain in their current situation than a comparison group of patients treated with physiotherapy for (painful) mobility complaints [44]. They also reported more pain and poorer physical functioning than patients with pituitary disease [45]. Physical, social, and role functioning, as well as pain and perceived physical and mental health were consistently worse in SCCH patients than in healthy controls [45]. Illness perceptions were comparable to those of patients with asthma and type 2 diabetes, with various significant differences which indicated that patients were aware of important aspects of their particular illnesses.

We have demonstrated significant associations between data reported about the pre-diagnostic period and responses concerning the situation at the time of the interview. Strong perceptions and feelings about one's health (i.e. PTS symptoms, despair, drama, concern, fear, and depression) in the pre-diagnostic period are related to negative health apperceptions in the present time.

Diagnostic delay was found to be negatively related to social support and positively related to minimum pain. In a recent publication [3] we established that, when controlling for age, diagnostic delay was also positively related to maximum pain, affective pain rating (PRIA), the BIPQ scales emotions and consequences, and unemployment/disability. Diagnostic delay was also negatively related to the SF20-scales role fulfillment and social functioning, and hours worked per week.

Because our study is retrospective with regard to the pre-diagnostic period, which for some patients occurred many years previously, it is possible that some or many respondents had no accurate recall of their physical and psychological conditions at that stage. This could represent two types of problems. First, the responses to the pre-diagnostic questions may contain random components that could be relatively large, leading to low reliability and lower correlations (attenuation). Since we obtained many sizeable correlations among the pre-diagnostic variables and between the latter and some of the present-day variables, this possible attenuation problem has not prevented us from finding some interesting relationships. However, it is still possible that unreliability may have precluded the discovery of additional interesting or important relationships. A second possibility is that respondents colored their responses to the pre-diagnostic questions on the basis of their present feelings and cognitions. Patients who felt better (worse) now might tend to report more (un)favorably about their past condition. In that case we would expect many (very) high correlations among most of the variables concerning the pre-diagnostic period. Indeed, we did find several high correlations, for example, *within *the scales measuring PTS and *within *those measuring social support, but not *between *those sets of variables. We also found that feelings of worry, fright, drama, concern, and depression could be reduced to one dimension, but this dimension did not have extremely high correlations with other pre-diagnostic variables. We can therefore conclude that potential retrograde coloring of responses did not seriously influence our results. We would like to stress that prospective research into the effects of pre-diagnostic conditions on later illness experience is practically impossible in the case of rare diseases such as SCCH.

As the pre-diagnostic variables were measured simultaneously with the present-day variables, we cannot be certain that the associations found between both sets imply that the present-day condition is caused or influenced by the pre-diagnostic situation. Nevertheless, the arguments presented above and the general results suggest that such an influence is probable.

## Conclusions

SCCH is an illness with serious consequences both in the pre-diagnostic period and after the diagnosis is established. SCCH patients reported relatively strong PTS symptoms, several unfavorable illness and health status perceptions, impaired quality of life, and considerable pain. Moreover, negative psychological states and lack of social support in the pre-diagnostic interval predict negative feelings and cognitions long after the diagnosis has been established. This implies that health care professionals should be alert for stress and other psychological problems of patients with unexplained chronic complaints as long as a diagnosis is not established, and that extensive efforts should be made to shorten this period.

## Competing interests

The authors declare that they have no competing interests.

## Authors' contributions

WAVDK initiated and coordinated the study. NATH, AAK, WAVDK, AAPVE, and LCSH are responsible for its design. SAC, LCSH, and FMCDD collected the data. SAC, LCSH, FMCDD and WAVDK analyzed the data. WAVDK drafted the manuscript and all authors contributed to its critical revision. All authors read and approved the final version.

## Authors' information

WAVDK is presently the chairman of the Netherlands Association of Patients with Sternocostoclavicular Hyperostosis.
